# Multi-Modal Integration of EEG-fNIRS for Characterization of Brain Activity Evoked by Preferred Music

**DOI:** 10.3389/fnbot.2022.823435

**Published:** 2022-01-31

**Authors:** Lina Qiu, Yongshi Zhong, Qiuyou Xie, Zhipeng He, Xiaoyun Wang, Yingyue Chen, Chang'an A. Zhan, Jiahui Pan

**Affiliations:** ^1^School of Software, South China Normal University, Guangzhou, China; ^2^Department of Rehabilitation Medicine, Zhujiang Hospital, Southern Medical University, Guangzhou, China; ^3^Guangdong Work Injury Rehabilitation Hospital, Guangzhou, China; ^4^School of Biomedical Engineering, Southern Medical University, Guangzhou, China

**Keywords:** multi-modality, electroencephalogram (EEG), functional near-infrared spectroscopy (fNIRS), brain activity, preferred music

## Abstract

Music can effectively improve people's emotions, and has now become an effective auxiliary treatment method in modern medicine. With the rapid development of neuroimaging, the relationship between music and brain function has attracted much attention. In this study, we proposed an integrated framework of multi-modal electroencephalogram (EEG) and functional near infrared spectroscopy (fNIRS) from data collection to data analysis to explore the effects of music (especially personal preferred music) on brain activity. During the experiment, each subject was listening to two different kinds of music, namely personal preferred music and neutral music. In analyzing the synchronization signals of EEG and fNIRS, we found that music promotes the activity of the brain (especially the prefrontal lobe), and the activation induced by preferred music is stronger than that of neutral music. For the multi-modal features of EEG and fNIRS, we proposed an improved Normalized-ReliefF method to fuse and optimize them and found that it can effectively improve the accuracy of distinguishing between the brain activity evoked by preferred music and neutral music (up to 98.38%). Our work provides an objective reference based on neuroimaging for the research and application of personalized music therapy.

## Introduction

Music is the reproduction of the sound of nature that combines science and art. It can not only be used as a form of entertainment to improve people's quality of life (Murrock and Higgins, 2010; Niet et al., 2010; Witte et al., 2020; Chen et al., 2021), but also as a treatment to cure some neurological diseases, such as Alzheimer's disease (Reschke-Hernández et al., 2020), stoke (Sarkamo et al., 2008), disorders of consciousness (DOC) (Carrière et al., 2020), Parkinson's disease (Alfredo, 2015), depression (Chen et al., 2021) and autism (James et al., 2015). For example, Reschke-Hernández et al. (2020) used familiar music as a stimulus to emphasize the effect of familiar music in inducing the emotions of patients with Alzheimer's disease. Sarkamo et al. (2008) showed that for stroke patients, listening music enhances cognitive recovery and mood after middle cerebral artery stroke. In their review, Rollnik and Eckart (2014) also concluded that listening to music has the effect of awakening and improving mood in patients with impaired consciousness, and can even be used as a means of communicating with patients.

With the rapid development of neuroimaging technology, more and more studies have focused on exploring the relationship between music and its effects on the brain. Previous studies have shown that music affects people not only psychological, but also has a positive impact on cognitive development of the brain, including memory, learning and attention (Franco Jarava, 2018). Bennet and Bennet (2008) found that listening to music helps keep brain neurons active and vigorous and synapses intact. Gui et al. (2019) described the use of fMRI to analyze the brain activity of depressed patients and healthy people under positive and negative music stimulation, and found that their regions of interest (ROI) characteristics are quite different. According to the functional near-infrared spectroscopy (fNIRS) indicator of the prefrontal cortex activation, Zheng et al. (2020) showed that soothing music cause low motivational intensity emotion and uplifting music cause high motivational intensity. In particular, music therapy with personality characteristics helps to make people feel relaxed and improve the mood, behavior and prognosis of patients (Zheng et al., 2020). Jagiello et al. (2019) used electroencephalogram (EEG) to measure the brain responses to familiar vs. unfamiliar music and found that fragments of familiar and unfamiliar music can be quickly distinguished in the brain.

Different states of the brain understand music in different ways and stimulate specific areas of the brain. Different genres of music have been shown to have different effects on brain function, such as significant differences in the activation of the prefrontal lobe were studied with classical and techno music (Bigliassi et al., 2015). Previous studies have shown that music with personal meaning may be more easily perceivable than background music or “relaxing” music, and it is more beneficial to people (Gerdner, 2000; Geethanjali et al., 2018; Jagiello et al., 2019). The findings of Greenberg et al. (2015) showed that individual differences in musical preferences may be related to brain activity and structure. Koelsch (2015) mentioned that because music can evoke people's memory, listening to familiar music can make the brain area which is responsible for memory function responds accordingly and induces people's emotions. Geethanjali et al. (2018) found specific activation and increased functional connectivity after listening to Indian music and Indian music can increased the positive affective scores. However, there are relatively few studies on the effect of personal preferred music on brain activity. What is the characteristic pattern of the brain activity induced by personal preferred music and whether it can promote our brain activity better than other audio stimulus are still unclear.

The current exploration of the relationship between music and brain activity mainly uses single-modality neuroimaging technology, such as fMRI, Positron Emission Computed Tomography (PET) and Magnetoencephalography (MEG) (Blood et al., 1999; Jared et al., 2018; Gui et al., 2019; Carrière et al., 2020). However, previous studies have proved that combining multi-modal imaging technology can effectively use the complementary information of different technologies to overcome the basic limitations of individual modalities, and provide more comprehensive and richer brain information than single-modality imaging technology (Cicalese et al., 2020). Among the commonly used non-invasive neuroimaging technologies, EEG and fNIRS are relatively portable, flexible and inexpensive, and have a wider range of possible applications. EEG can capture the macro-time dynamics of brain electrical activity by recording neuron firing (Pan et al., 2020), and fNIRS estimates brain hemodynamic fluctuations through spectral measurement (Chincarini et al., 2020). These two technologies reflect different aspects of brain neural activity. In addition, EEG measurement has high time resolution but poor stability, while fNIRS has higher spatial resolution and good anti-interference but lower time resolution (He et al., 2020). Therefore, the multi-modal brain imaging system that combining both EEG and fNIRS can simultaneously obtain high-temporal-spatial resolution information, and dynamically observe the information processing processes of the cerebral cortex from the two dimensions of neuroelectric activity and hemodynamics. This is undoubtedly a better strategy for exploring brain activity. Li et al. (2020a) developed an EEG-informed-fNIRS analysis framework to investigate the neuro-correlate between neuronal activity and cerebral hemodynamics by identifying specific EEG rhythmic modulations which contribute to the improvement of the fNIRS based General Linear Model (GLM) analysis. Putze et al. (2013) used EEG and fNIRS to distinguish and detect visual and auditory stimuli processing. They extracted the time-domain and frequency-domain features of EEG and the mean feature of fNIRS and then used the classifier to classify them. They concluded that the fusion of different features of different modal signals has more advantages than the classification accuracy of single-modality features. The multi-modal EEG-fNIRS can provide richer brain activity information than a single-modality. Inspired by the above research, we tried to integrate the multi-modal brain imaging technology of EEG and fNIRS to explore brain activity evoked by personal preferred music.

However, EEG and fNIRS signals are two different brain signals, and the multi-modal integration of EEG-fNIRS is still a challenge in the field of multi-modal research. The commonly used multi-modal integration methods mainly include three strategies: data-level fusion, feature-level fusion, and decision-level fusion. Among them, the feature-level fusion strategy with relatively good effect and relatively simple processing has attracted more attention (Qi et al., 2018). However, most of the current researches using this strategy simply use feature vector splicing to fuse multi-modal features (Hubert et al., 2017). Although the splicing method is simple, it does not consider the correlation and difference between different modalities, and it is difficult to utilize the multi-modal information of EEG-fNIRS fully and effectively. An effective multi-modal integration method can further improve the performance of the EEG-fNIRS system (Khan and Hasan, 2020).

In this work, we propose a multi-modal integration framework of EEG-fNIRS from data collection to data analysis to explore the influence of personal preferred music on brain activity. Under the stimuli of personal preferred music and neutral music, we synchronously collected the brain signals of the two modalities, and combined their features to explore the brain activities evoked by music. Meanwhile, we employed an improved Normalized-ReliefF algorithm to fuse and optimize the multi-modal features from the two brain signals, which effectively improves the accuracy of distinguishing brain activity caused by preferred music and neutral music. The main contributions of this work are as follows:

1. Based on the complementarity of EEG and fNIRS, multi-modal data of EEG and fNIRS were simultaneously collected to explore the relationship between music (especially favorite music) and brain activity from different perspectives, which provides imaging-based evidence for the clinical application of personalized music therapy.
2. A multi-modal integration method of EEG-fNIRS is proposed. We first normalized the multi-modal features from EEG and fNIRS, and then developed an improved ReliefF algorithm to perform feature selection on these multi-dimensional features, and finally fused these features together to realize the full utilization and effective fusion of EEG and fNIRS information.

## Materials and Methods

### Subjects

Nine right-handed volunteers (five males and four females, with an average age of 31.25 years) with no history of neurological, psychiatric or other brain-related dis-ease participated in this study. No subjects reported damage to the auditory channel or received professional musical education. Before the start of the experiment, each subject was fully informed of the experimental purpose and methods, and provided writ-ten informed consent prior to the start of the experiment.

### Paradigm Design

**Figure 1:**
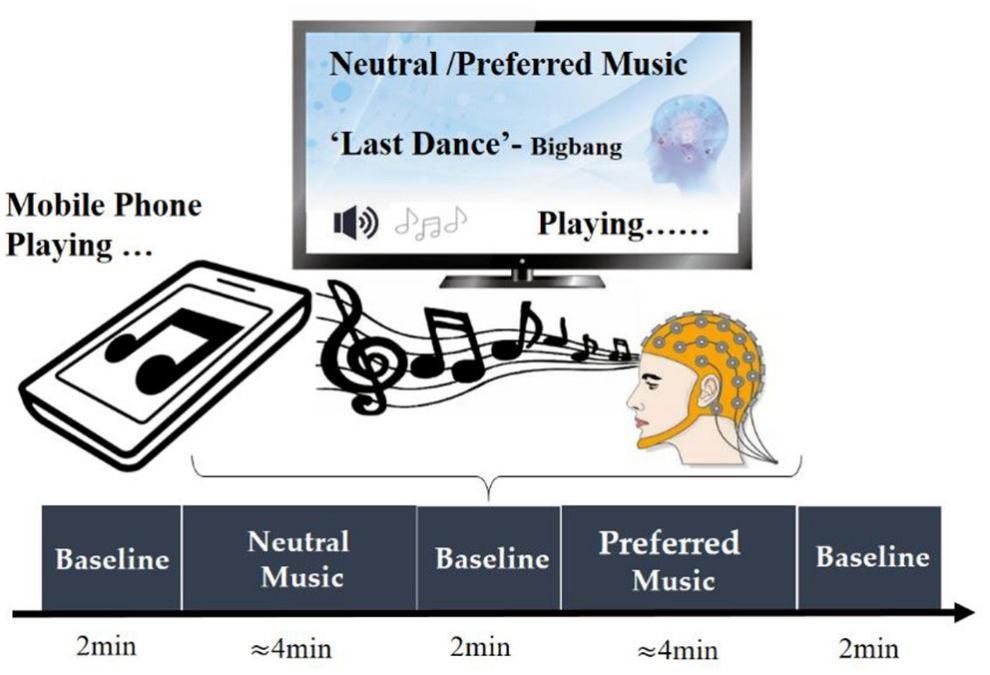
The paradigm design of this experiment.

Participants' personal preferred and neutral music was used as the experimental stimulus in the present study. Before the experiment, we conducted a questionnaire survey for each participant, asking them to provide one of their personal favorite music, and choosing one of the four unfamous relax music (e.g., soft music) we provided as the neutral music stimulus. All music has lyrics. During the experiment, music was played outside via mobile phones, and the playback volume of each song was basically the same. In order to minimize the interference of environmental noise, we kept the experimental environment as quiet as possible. At the beginning of the experiment, the subject was asked to close their eyes and sit awake in a comfortable chair. Short beeps were emitted at the beginning and end of music playback to indicate the beginning and end of the music stimulus. At the beginning of the measurement, the subjects were asked to stay relaxed for 2 min, and then performed neutral music stimulus task, that is, to continuously listen to a piece of neutral music (about 4 min). After the stimulus task of neutral music, the subjects were asked to stay relaxed for 2 min, and then performed the stimulus task of personal preferred music, that is, listen to a continuous music that the subjects are favorite (about 4 min). After the stimulus task of personal preferred music, the subjects remained at rest for 2 min before ending the experiment. The specific process of the experimental paradigm is shown in Figure 1.

### Data Collection

**Figure 2:**
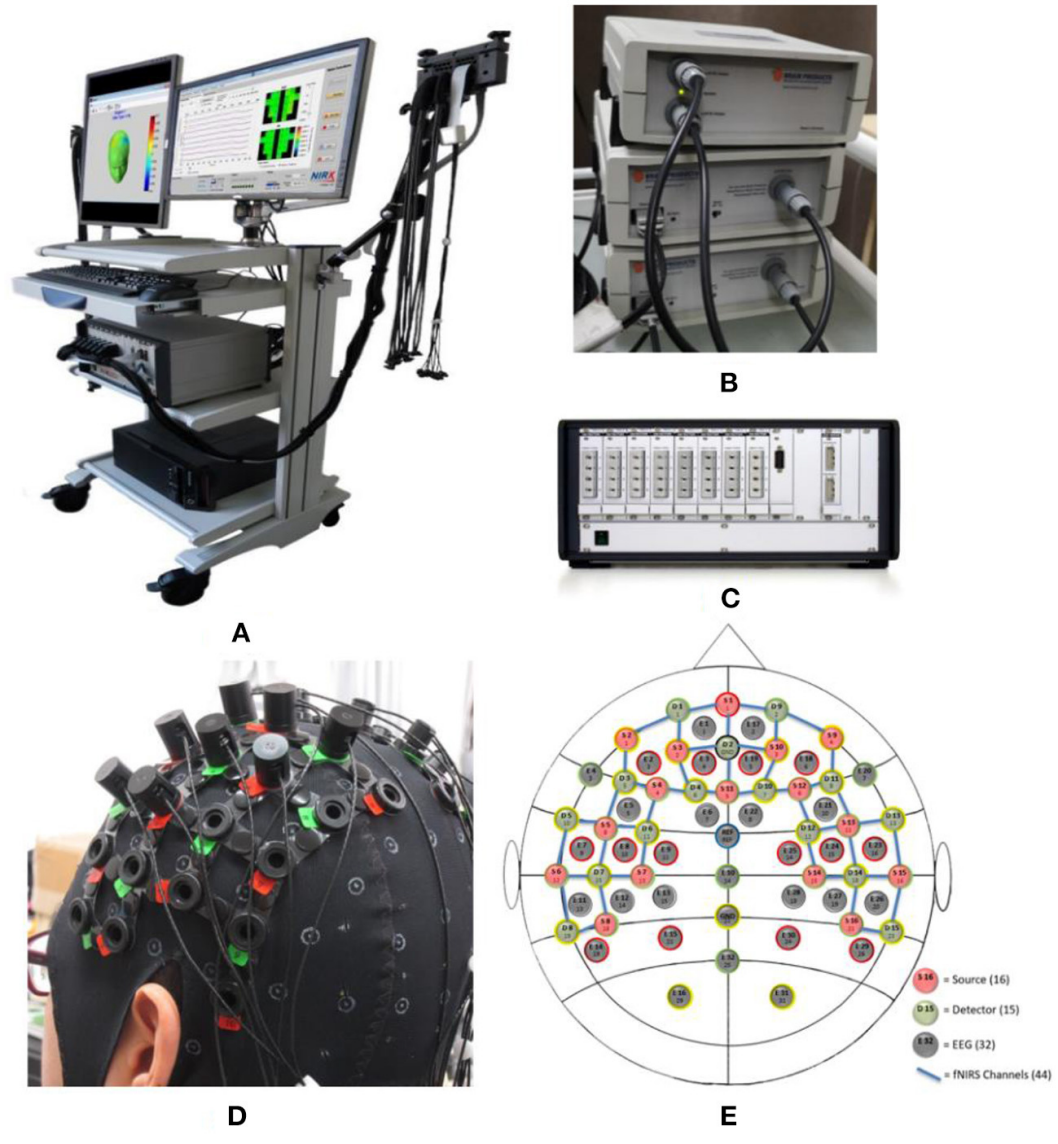
Acquisition equipment and measuring cap. **(A)** View of the acquisition equipment; **(B)** Acquisition amplifier of EEG; **(C)** Laser light source of fNIRS; **(D)** View of measuring cap; and **(E)** Channel location of EEG and fNIRS. Where the red circles represent the 16 light sources of fNIRS, the green circles are 15 detesctors of fNIRS, the blue lines are 44 channels of fNIRS, and the gray circles represent 32 EEG electrodes.

In this study, we first used EEG technology alone to collect the brain signals of four subject listening to neutral music and their personal preferred music. After preliminary analysis confirmed that the two kinds of music did have an effect on brain activity, we then used EEG and fNIRS technology to simultaneously collect data from five other subjects. EEG signals were collected using a BrainAmp DC EEG recording system (Brain Products GmbH, Germany). The electrode placement follows the international 10–20 convention of a 32-channel cap, and the signal was recorded at a sampling rate of 500 Hz. The fNIRS signals were recorded using a multi-channel NIRScout system (NIRx Medizintechnik GmbH, Germany) at a sampling rate of 3.91 Hz. The source-detector distance was fixed at 3 cm, and a total of 44 measurement channels. The EEG-fNIRS acquisition equipment and the EEG and fNIRS channel locations is shown in Figure 2. During the data collection, we used a computer and E-Prime software to form a signal prompting device. We connected the mobile phone to the computer via a USB data cable, and the computer controls the mobile phone to play music, so as to achieve the purpose of controlling the signal prompt device and the mobile phone to play music synchronously. The signal prompting device sends a trigger signal to BrainAmp DC EEG recording system and NIRScout system at the same time through the parallel port. BrainAmp DC EEG recording system and NIRScout system, respectively, amplify these two kinds of brain signals. Finally, the computer simultaneously recorded the brain signals and the event markers processed by the two system.

### Data Processing

#### EEG-fNIRS Data Preprocessing

EEG preprocessing was performed using EEGLAB software (v2021.0). Data was first re-referenced to a common-average reference and then filtered from 0.5 to 50 Hz. In order to maintain the consistency of the data of each subject, we kept the 200-s climax part of the two pieces of music, so the EEG data of each piece of music were segmented to form a period of data from 5 to 205 s after the start of the stimulus. Then, a baseline correction was performed on the segmented data of each stimulus. Finally, independent component analysis (ICA) was used to remove the ocular artifact for each subject.

fNIRS preprocessing was performed using nirsLAB software (v201904). First, a 4th order Butterworth band-pass filter with cut-off frequencies of 0.01–0.1 Hz, was applied to remove artifacts such as those originated from heartbeats (~1 Hz), venous pressure waves due to respiration (~0.2 Hz) and arterial pressure oscillations (Mayer waves ~0.1 Hz). Then, the concentration changes of oxyhemoglobin (*HbO*) and deoxyhemoglobin (*Hb*) were computed according to the Modified Beer-Lambert Law. In fNIRS, *HbO* and *Hb* are the parameters that can indirectly reflect the neural activity of the brain, and are often used in fNIRS data analysis (Yücel et al., 2021). Then, the baseline correction of each channel of the fNIRS signals were performed by subtracting the mean value of the 10 s baseline signal before the start of the stimulus from the signal during each stimulus task. Finally, the fNIRS data of each piece of music were also segmented to form a period of data from 5 to 205 s after the start of the stimuli.

#### Feature Extraction

- **EEG signals:** We extracted time domain, frequency domain, time-frequency domain and spatial domain features to analyze the characteristics of brain activity.
  - **Time Domain Features**
    Regarding the feature extraction in time domain, and we used centralized statistical methods to represent the time series of EEG (Li et al., 2020b). These time-domain statistic features include:
    **Mean:**
    (1) $$\begin{array}{r} \mu_{s}=\frac{1}{N}\overset{N}{\underset{i=1}{\sum }}S\left(i\right) \end{array}$$
    **Standard deviation:**
    (2) $$\begin{array}{r} \sigma_{s}^{2}=\frac{1}{N}\overset{N}{\underset{i=1}{\sum }}{\left[S\left(i\right)-\mu_{s}\right]}^{2} \end{array}$$
    **Mean of the 1st difference absolute value:**
    (3) $$\begin{array}{r} \delta_{s}=\frac{1}{N-1}\overset{N-1}{\underset{i=1}{\sum }}|S\left(i+1\right)-S\left(i\right)| \end{array}$$
    **Mean of the normalized 1st difference absolute value:**
    (4) $$\begin{array}{r} \bar{\delta_{s}}=\frac{1}{N-1}\overset{N-1}{\underset{i=1}{\sum }}|\bar{S}\left(i+1\right)-\bar{S}\left(i\right)|=\frac{\delta_{s}}{\sigma_{s}} \end{array}$$
    **Mean of the 2nd difference absolute value:**
    (5) $$\begin{array}{r} \gamma_{s}=\frac{1}{N-2}\overset{N-2}{\underset{i=1}{\sum }}|S\left(i+2\right)-S\left(i\right)| \end{array}$$
    **Mean of the normalized 2nd difference absolute value:**
    (6) $$\begin{array}{r} {\bar{\gamma }}_{s}=\frac{1}{N-2}\overset{N-2}{\underset{i=1}{\sum }}|\bar{S}\left(i+2\right)-\bar{S}\left(i\right)|=\frac{\gamma_{s}}{\sigma_{s}} \end{array}$$
    where *i* is the sampling point, *S*_*i*_ represents the EEG signals, *N* is the number of samples. Then, we put the above statistical features of the signals into a vector as follows:
    (7) $$\begin{array}{r} FV_{statistic}=\left[\mu_{s},\sigma_{s}^{2},\delta_{s},\bar{\delta_{s}},\gamma_{s},{\bar{\gamma }}_{s}\right] \end{array}$$
  - **Frequency Domain Features**
    For frequency domain features, we extract the two most typical features: PSD feature (Åkerstedt and Gillberg, 1986) and DE feature (Duan et al., 2013).
    **PSD** is a measure of the mean square value of a random variable, and it is the average power dimension per unit frequency. The average power of the signal can be obtained by integrating the power spectrum in the frequency domain. We used the periodogram method (Meziani et al., 2019) to obtain the power spectral density, and we calculated the PSD of the five frequency bands [δ (0.5–3 Hz), θ (4–7 Hz), α (8–13 Hz), β (14–30 Hz), and γ (30–50 Hz)]. Periodogram is a simple and popular method of spectrum estimation, which is based on Discrete Fourier Transform (DFT):
    (8) $$\begin{array}{r} F\left[k\right]=\;\overset{N-1}{\underset{n=0}{\sum }}x\left[n\right]e^{-\frac{j2\pi kn}{N}} \end{array}$$
    where *Fs* is the sampling rate of the EEG signals, *j* and π are constants, and *N* is the number of samples, n is the sampling point, *k* = 0, 1, 2…*N* − 1. We can get the periodic diagram of a discrete-time signal *x*[*n*], *n* = 1, 2, …, *N* with a sampling rate of *Fs* is calculated as:
    (9) $$\begin{array}{r} p\left(f\right)=\;\frac{1}{NF_{s}}{\left|F\left[k\right]\overset{N}{\underset{k=1}{\sum }}w\left[k\right]\right|}^{2} \end{array}$$
    where *f* = *kFs*/*N* and *p*(*f*) is the PSD feature of the EEG.
    **DE** is the generalized form of Shannon entropy (ShEn) $S_{en}=\sum_{i=1}^{n}p\left(s_{i}\right)\log_{a}\frac{1}{p\left(s_{i}\right)}=-\sum_{i=1}^{n}p\left(s_{i}\right)\log_{a}p\left(s_{i}\right)$ on continuous variables:
    (10) $$\begin{array}{r} DE=\;\underset{a}{\overset{b}{\int }}S_{en}dx=-\underset{a}{\overset{b}{\int }}p\left(s_{i}\right)\log\left[p\left(s_{i}\right)\right]ds_{i} \end{array}$$
    where *p*(*s*_*i*_) represents the probability density function of continuous information, [*a, b*] represents the interval of information value.
  - **Time-Frequency domain**
    **Wavelet Entropy** is the entropy value calculated by the wavelet transform of the signal according to its probability distribution (Quiroga et al., 2001). Shannon's theory of entropy provides a useful tool for analyzing and comparing probability distributions. The calculation formula of wavelet entropy is as follows:
    (11) $$\begin{array}{r} S_{WT}=S_{WT}\left(p\right)=-\underset{i}{\sum }p_{i}\text{ln }\left[p_{i}\right] \end{array}$$
    where *p* represents the energy intensity ratio of a certain signal.
  - **Spatial Features**
    The spatial features of EEG signals generally refer to the combined features obtained by comparing the signal features of left-right symmetrical area by using the spatial position information of the EEG electrodes. The spatial domain features are based on the principle that different brain activity states have different activation levels in different areas of the brain. The spatial domain features are calculated as follows: Time domain, frequency domain, and time-frequency domain features of the EEG signals of each channel are used as preliminary features, and then the left-right symmetric electrode feature combination is used as the final spatial domain features. The placement of the 32 EEG electrodes used in our experiment corresponds to the position on the international 10–20 convention. The Cz and Pz in the middle position are removed, and the remaining 30 channel electrodes from 15 symmetrical left-right electrode (AFp1-AFp2, AFF1h-AFF2h, AFF5h-AFF8h, F7-F8, FFC5h-FFC8h, FFC1h-FFC2h, FCC3h-FCC4h, FCC5h-FCC8h, FTT7h-FTT8h, CCP3h-CCP6h, CCP5h-CCP6h, TTP7h-TTP8h, CPP3h-CPP4h, TPP7h-TPP8h, PO3-PO4, a total of 15 pairs of electrodes).
    **RASM** refers to the ratio of the eigenvalues of the symmetrical electrode pair on the left and right (Li et al., 2020b). We used the symmetrical electrode feature values (*FX*_*L*−*EEG*_, *FX*_*R*−*EEG*_) of the left and right brain regions to obtain RASM features. The calculation formula of RASM is as (12):
    (12) $$\begin{array}{r} F_{RASM}=\frac{FX_{L-EEG}}{FX_{R-EEG}} \end{array}$$
- **fNIRS signals:** We analyzed the features of the fNIRS signals from the perspective of time domain (Naseer and Hong, 2015), considering the features of the change of *HbO* concentration and the change of *Hb* concentration (we denoted it as and), including their statistic features and features based on GLM.
  - **Statistic Features**
    We extracted the mean and variance the *HbO* and *Hb* signals of 44 channels of the fNIRS signals as statistical features. The calculation formulas of the two statistic features are shown in (13) and (14):
    (13) $$\begin{array}{r} \mu_{f}=\;\frac{\sum_{n=t_{1}}^{t_{2}}|x\left(n\right)|}{f_{s}\left(t_{2}-t_{1}\right)} \end{array}$$
    (14) $$\begin{array}{r} \sigma_{f}^{2}=\frac{\sum_{i=t_{1}}^{t_{2}}{\left[x{\left(n\right)}^{\prime }-\mu_{f}\right]}^{2}}{f_{s}\left(t_{2}-t_{1}\right)} \end{array}$$
    where *x*(*n*) are the and signals, *t*_1_ and *t*_2_ (*t*_2_ > *t*_1_) are two time points, μ_*f*_ and $\sigma_{f}^{2}$ are the mean and the variance value of the fNIRS signals in the time periods *t*_1_ and *t*_2_. We combine the above two statistical features into a feature vector as the statistical feature *SF*_*f*_ of the fNIRS signals.
    (15) $$\begin{array}{r} SF_{f}=\left[\mu_{f},\sigma_{f}^{2}\right] \end{array}$$
  - **Feature based on GLM**
    When the brain activation changes, *HbO* usually exhibits an approximately linear trend. Using GLM, the *B* value representing the degree of activation of each channel can be calculated to detect the activated channel. The calculation formula of GLM of fNIRS signals is:
    (16) $$\begin{array}{r} Y=BX+E \end{array}$$
    In formula (15), *Y* is the preprocessed *HbO* data or *Hb* data of the fNIRS signals as the dependent variable, and *X* is a design matrix. *E* is a residual matrix that obeys a normal distribution, and *B* is a matrix with estimated parameters. Based on formula (16), each item of the matrix *Y* is *y*_*ij*_, *i* = 1, 2, …, *N* represents the number of time points of data acquisition, and *j* = 1, 2, …, *N* represents the number of channels. That is, *y*_*ij*_ is the *HbO* data or *Hb* data collected by the *j*^*th*^ channel at the *i*^*th*^ time point. Therefore, when each item of *Y* is *y*_*ij*_, we can get the calculation formula of *y*_*ij*_ as:
    (17) $$\begin{array}{r} y_{ij}=x_{i1}\beta_{1j}+x_{i2}\beta_{2j}+\ldots +x_{ik}\beta_{kj}+\varepsilon_{ij} \end{array}$$
    Then we can transform formula (17) into:
    (18) $$\begin{array}{r} \left[\begin{array}{c} y_{1}\\ y_{2}\\ \vdots \\ y_{m} \end{array}\right]=\left[\begin{array}{cccc} x_{11} & x_{12} & \ldots & x_{1n}\\ x_{21} & x_{22} & \ldots & x_{2n}\\ \vdots & \vdots & \ddots & \vdots \\ x_{m1} & x_{m2} & \ldots & x_{mn} \end{array}\right]\left[\begin{array}{c} \beta_{1}\\ \beta_{2}\\ \vdots \\ \beta_{n} \end{array}\right]+\left[\begin{array}{c} \varepsilon_{1}\\ \varepsilon_{2}\\ \vdots \\ \varepsilon_{n} \end{array}\right] \end{array}$$
    Finally, we can obtain the feature *B* value of fNIRS signals by the least square method:
    (19) $$\begin{array}{r} B={\left(X^{\prime }X\right)}^{-1}X^{\prime }Y={\left[\beta_{1}\beta_{2}\ldots \beta_{k}\right]}^{T} \end{array}$$

#### Feature Fusion

Since EEG data and fNIRS data are two different brain signals, they are quite different in principle and acquisition mechanism. When combining these two different types of data, simple feature splicing often leads to poor performance of the machine learning algorithm. Therefore, it is very necessary to normalize the features of the two before fusion and perform feature selection after fusion. Based on this, we proposed a multi-modal feature-level fusion method, the Normalized-ReliefF method. Due to the amplitude and dimension of the various features of the two brain signals are different, in order to more effectively fuse them, we used the normalization algorithm of formula (20) to modulate all the features so that they were scaled to the range of 0 to 1. The normalized features were fused into a new multi-modal feature vector *Mul*_*Feat*_, as in formula (21).

(20) $$\begin{array}{r} Fea{t_{i}}^{\prime }=\;\frac{Feat_{i}-min\left(Feat_{i}\right)}{\max\left(Feat_{i}\right)-min\left(Feat_{i}\right)} \end{array}$$

(21) $$\begin{array}{r} Mul_{Feat}=\left[Fea{t_{1}}^{\prime }\ldots Fea{t_{i}}^{\prime }\right] \end{array}$$

Considering that there may be redundant information between these between these multi-modal features, it is often difficult to manually extract complementary and non-redundant information. Therefore, we further used the ReliefF algorithm to optimize the selection of features to achieve more efficient fusion of the two signals. ReliefF is based on the ability of features to distinguish close samples of each class, and evaluates the features by assigning different weights to the features. The larger the feature weight, the more helpful it is to distinguish the categories. When the correlation between the feature and the classification is extremely low, the weight of the feature will be very small, even close to 0. The feature weight may be negative, which means that the distance between similar neighboring samples is greater than the distance between different types of neighboring samples, that is, the feature has a negative impact on classification. For the sample set *Q*, a sample S is randomly selected each time, and then k neighboring samples *NH* of *S* are searched for in the same sample set of *S*, and k neighboring samples *NM* are searched for each sample set of a different category from *S*. Iteratively update the weight ω(*x*) of each feature, and the update formula is:

(22) $$\begin{array}{r} \omega {\left(x\right)}^{\prime }=\frac{\begin{array}{c} \sum_{j=1}^{k}diff\left(X,S,NH_{j}\right)+\sum_{C\neq Cl\left(s\right)}\\ \left[\frac{P\left(C\right)}{1-P\left[Cl\left(S\right)\right]}*\sum_{j=1}^{k}diff\left[X,S,NM{\left(C\right)}_{j}\right]\right] \end{array}}{m*k} \end{array}$$

(23) $$\begin{array}{r} \omega \left(x\right)=\omega \left(x\right)-\omega {\left(x\right)}^{\prime } \end{array}$$

(24) $$\begin{array}{r} diff\left(X,S,S^{\prime }\right)=\{\begin{array}{c} \frac{\left|S\left[X\right]-S^{\prime }\left[X\right]\right|}{\max\left(X\right)-min\left(X\right)},where\;X\;is\;continuous\\ 0,\;where\;X\;is\;discrete\;and\;S\left[X\right]=S^{\prime }\left[X\right]\\ 1,\;where\;X\;is\;discrete\;and\;S\left[X\right]\neq S^{\prime }\left[X\right] \end{array} \end{array}$$

In the formulas (22) and (24), *m* represents the number of iterations, *NH*_*j*_ defines the *j*^*th*^ nearest neighbor sample of the same class, and *NM*(*C*)_*j*_ is the *j*^*th*^ nearest neighbor sample of a different class of class *C* samples. *P*(*C*) represents the probability of the *C*^*th*^ target, *Cl*(*S*) refers to the category to which sample *S* belongs, *diff*(*X, S, S*′) is the distance between sample *S* and *S*′with respect to feature *X*.

#### Classification

The time domain, frequency domain and spatial domain features are extracted from the EEG signals of each subject, and the statistical features and features based GLM are extracted from fNIRS. Based on these features of EEG and fNIRS, we used six classifiers to classify subjects' brain activity induced by personal preferred and neutral music. The six classifiers we used are support vector machines (SVM), k-nearest neighbor (KNN), Random Forest, AdaBooting, Naive Bayesian and discriminant analysis classifiers (DAC). In order to better perform multi-modal signal fusion, we modulated the signal characteristics of these two different modalities into signals with the same sampling rate (1 Hz) through sample packaging before classification. Furthermore, we performed a 5-fold cross-validation for each classification in order to avoid the phenomenon of false high accuracy.

**SVM** is probably one of the most popular and watched machine learning algorithms. The hyperplane is the line that divides the input variable space. In SVM, the hyperplane is selected to best separate the points in the input variable space from their class (level 0 or level 1). The SVM learning algorithm finds the coefficients that make the hyperplane to best separate the classes.

**KNN** is a commonly used statistical classification method, which can be used not only for regression or linear classification, but also for non-linear classification. It can achieve high classification accuracy, has no assumptions about the data, and is not sensitive to outlier.

**Random Forest** is a classifier that contains multiple decision trees in machine learning, and the output category is determined by the modal of the category output by the individual tree.

**Naive Bayesian** is a series of simple probability classifiers based on the use of Bayes' theorem under the assumption of strong independence between features. The classifier model assigns class labels represented by feature values to the problem instances, and the class labels are taken from a limited set.

**AdaBoosting** is a kind of ensemble method classifier. Each time this method uses bootstrap sampling to construct a tree, it increases the sampling weight for the misjudged observations based on the results of the previous tree, so that the next tree can be more representative of the misjudged observations.

The basic idea of **DAC** is to project high-dimensional pattern samples into the best discriminant vector space to achieve the effect of extracting classification information and compressing the dimension of the feature space. After projection, it is ensured that the model samples have the largest inter-class distance and the smallest intra-class distance in the new subspace, that is, the model has the best separability in the space. Therefore, it is an effective feature extraction method. Using this method can maximize the inter-class scatter matrix of the pattern samples after projection, and at the same time minimize the intra-class scatter matrix.

**Figure 3:**
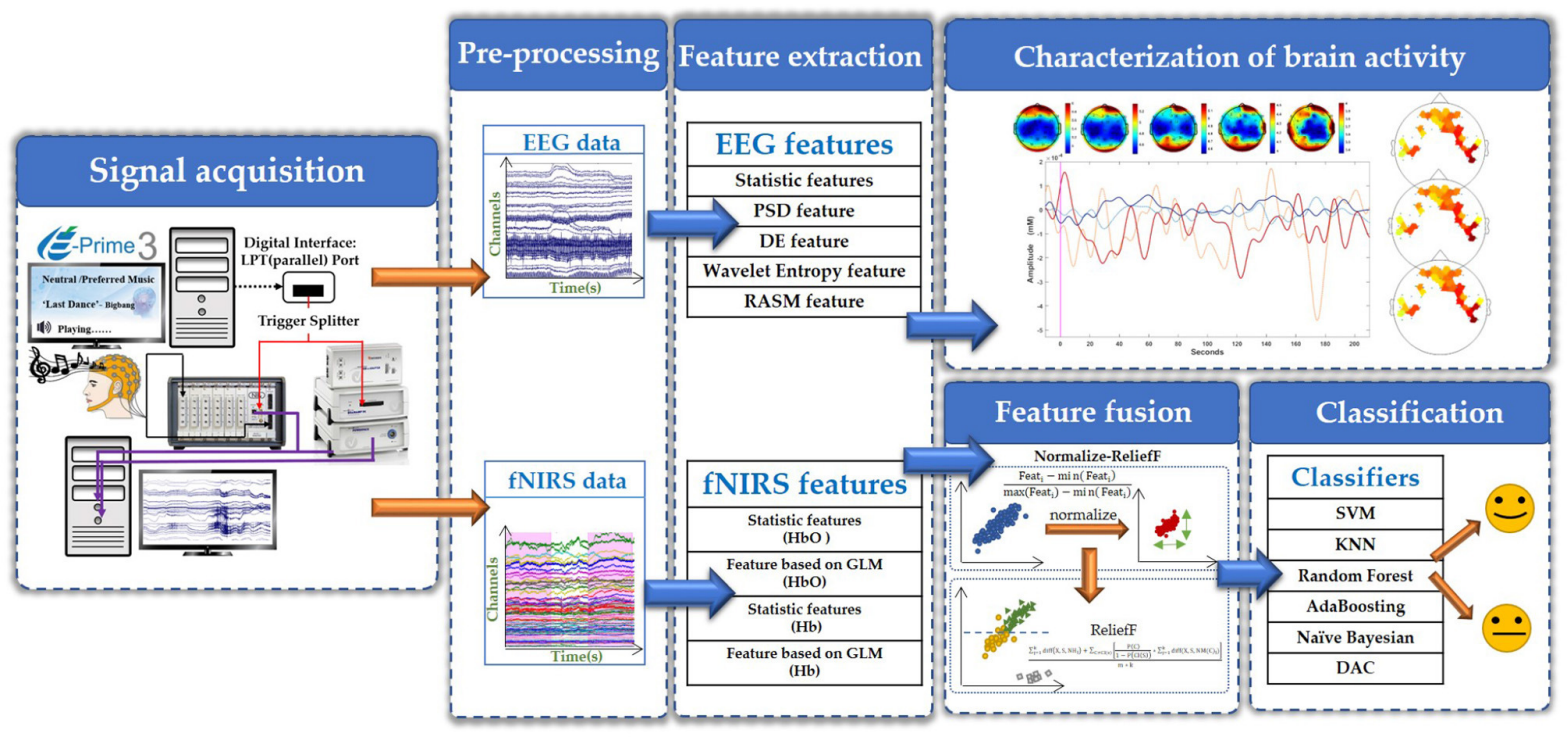
The overall framework of EEG-fNIRS multi-modal integration.

In summary, as shown in the overall framework of multi-modal EEG-fNIRS integration in Figure 3, we integrated EEG and fNIRS from data collection to data analysis to explore the characteristics of music on brain activity.

## Results

**Figure 4:**
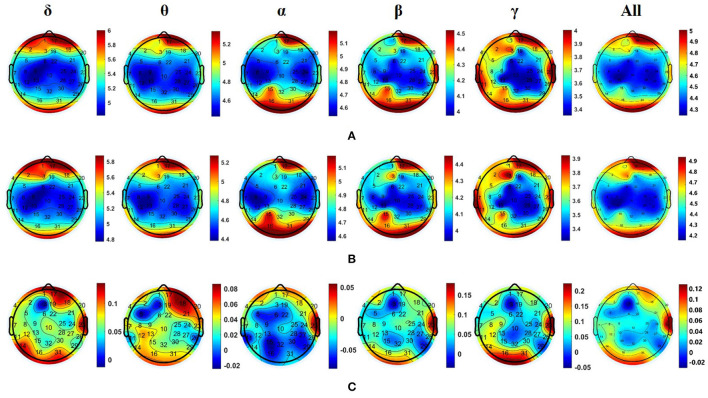
Averaged DE distributions of all nine subjects in the five frequency bands induced by personal preferred music **(A)** and neutral music **(B)**, and their DE difference distribution [i.e., personal preference minus neutral music, as shown in **(C)**] in the five frequency bands. Where δ: 0.5–3 Hz; θ: 4–7 Hz; α: 8–13 Hz; β: 14–30 Hz; γ: 30–50 Hz; All (0.5–50 Hz).

For EEG signals recorded by nine subjects while listening to neutral music and their personal preferred music, we calculated the DE values of the five common frequency bands (i.e., δ: 0.5–3 Hz, θ: 4–7 Hz, α: 8–13 Hz, β: 14–30 Hz, and γ: 30–50 Hz) of all channels and distribution of all nine subjects in the five frequency bands induced by personal preferred music and neutral music. Figure 4A is the average DE distribution diagram of personal preferred music, Figure 4B is the average DE distribution diagram of neutral music, and Figure 4C is the difference diagram of the average DE distribution of personal preferred music and neutral music (personal preferred music minus neutral music). It can be seen from Figure 4 that when the subjects listened to personal preferred music (A) and neutral music (B), the brain had a similar response pattern. The specific performance is as follows: firstly, the two kinds of music in each frequency band show a similar DE distribution pattern; secondly, personal preferred music and neutral music mainly activate the prefrontal lobe (especially the right frontal lobe) and the occipital lobe; finally, in the same brain area, the activation of the lower frequency band (e.g., δ and θ) is often stronger than that of the higher frequency band (e.g., β and γ). Moreover, we analyzed the differences in brain activity induced by the two music by subtracting the average DE of neutral music (B) from the average DE of personal preference music (A), as shown in Figure 4C. It can be seen that personal preferred music activates our brain more in right prefrontal, occipital and right temporal regions, and this difference is more obvious in the δ frequency bands.

**Figure 5:**
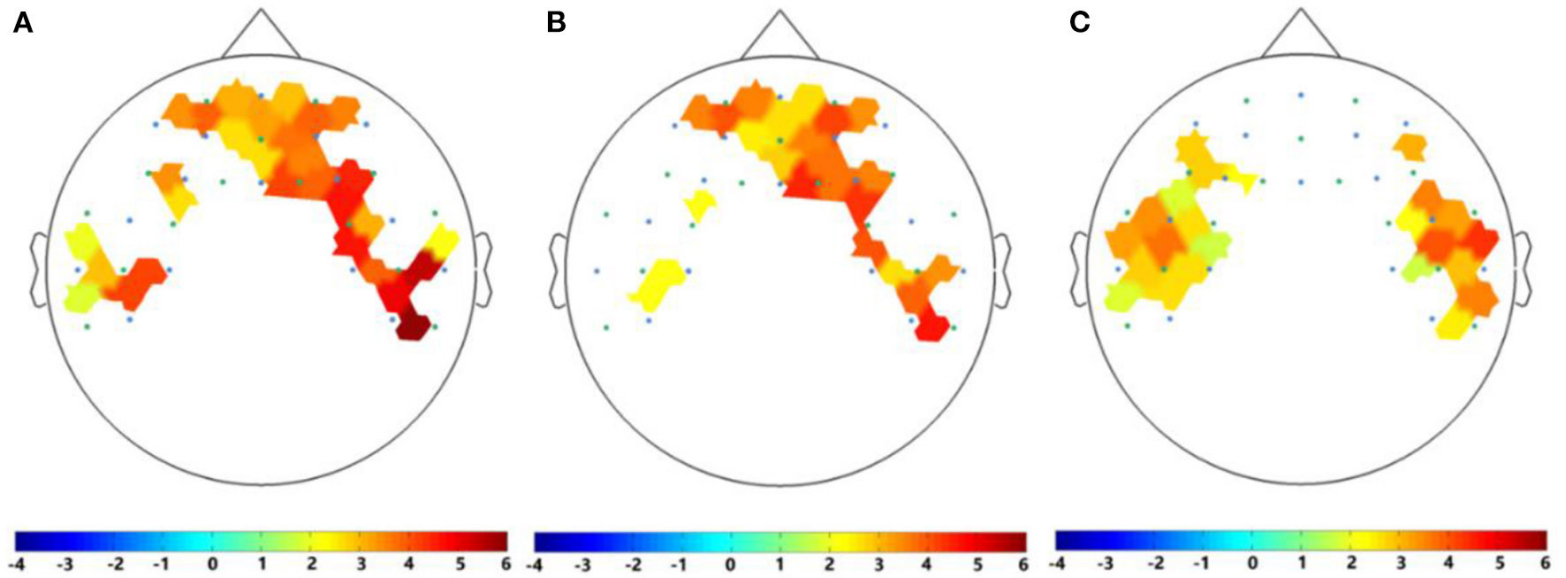
*HbO* of contrasts of **(A)** personal preferred music stimulus vs. baseline, **(B)** neutral music stimulus vs. baseline, and **(C)** personal preferred music vs. neutral music. The area shown in the figure represents a statistically significant difference (*p* < 0.05). Where the blue circles represent the light sources of fNIRS and the green circles are the detectors of fNIRS. The darker the red, the more significant the difference and the smaller the *p*-value.

In this study, due to the limited number of optodes of the fNIRS equipment, which cannot cover the entire human head, we used the high spatial resolution of fNIRS to distribute the 44 channels mainly in the prefrontal and left and right temporal lobes to collect signals. For the fNIRS signals recorded by five subjects while listening to neutral music and their personal preferred music, we calculated the changes in *HbO* concentrations in all 44 channels of each subject. We averaged the *HbO* concentration of all subjects and compared the *HbO* concentration during music listening with the *HbO* in the resting state. We found that compared with the resting state, personal preferred music and neutral music significantly enhanced brain activity. As shown in Figure 5, *HbO* concentration indicates that the brain response patterns induced by personal preferred music (A) and neutral music (B) are also similar, both of which are significantly (*p* < 0.05) activates the prefrontal lobe and part of the right temporal lobe activation. Moreover, the brain activation induced by personal preferred music is stronger than that of neutral music, and their differences are mainly manifested in the bilateral temporal lobes, as shown in Figure 5C. In this study, *t*-test2 was used for significance analysis. The brain areas shown in Figure 5 are statistically significant (*p* < 0.05).

In order to further explore the differences in brain activity induced by personal preferred and neutral music, we used six classifiers (i.e., SVM, KNN, Random Forest, AdaBoosting, Naïve Bayesian, and DAC) to classify the features of EEG and fNIRS under the personal preferred and neutral music. For EEG, the features used for classification include DE, PSD, Statistic, Wavelet Entropy, RASM and all EEG fused features. The classification results based on EEG features is shown in Table 1. It can be seen from Table 1 that most of the classifiers can effectively distinguish whether the subject is listening to personal preferred or neutral music based on the characteristics of brain activity. Among multiple features, the combined features have the best average classification effect in all classifiers, with an averaged accuracy of 93.45%. Among the six classifiers, the Random Forest classifier has the highest average classification accuracy, with an averaged accuracy of 86.66%.

**Table 1:** Classification accuracy of different classifiers based on EEG different features.

	**DE**	**PSD**	**Statistic**	**Wavelet entropy**	**RASM**	**Combined features**	**Averaged accuracy**
SVM	93.94%	94.32%	96.60%	49.06%	83.12%	97.17%	85.70%
KNN	80.59%	86.49%	91.00%	52.01%	70.34%	88.01%	78.07%
Random forest	92.12%	94.91%	95.69%	52.17%	87.41%	97.63%	86.66%
AdaBoosting	92.12%	94.91%	96.35%	50.77%	86.55%	97.94%	86.44%
Naive bayesian	78.49%	70.71%	80.81%	51.67%	82.01%	84.78%	74.75%
DAC	91.29%	90.11%	92.42%	49.12%	85.35%	95.14%	83.91%
Averaged accuracy	88.09%	88.58%	92.14%	50.80%	82.4%7	93.45%	

For fNIRS, we converted it into *HbO* and *Hb* through the modified Beer Lambert's law, then divided the data and extracted the statistical feature values and the feature values based on GLM of *HbO* and *Hb* per second. After that, we encapsulated the above four features at one sample per second, and statistic feature has a dimension of 400^*^280 and the feature based on GLM has a dimension of 400^*^140. We also used the above six classifiers to classify the four features of which are the statistic features and the feature based on GLM of *HbO* and *Hb*. As shown in Table 2, the accuracy of the statistical feature based on *Hb* classification under the KNN classifier is the highest, reaching 91.39%, and the statistic feature are better than the classification effect of the features extracted based on GLM.

**Table 2:** Classification accuracy of different classifiers based on fNIRS different features.

	**Statistic (HbO)**	**GLM (HbO)**	**Statistic (Hb)**	**GLM (Hb)**	**Averaged accuracy**
SVM	80.91%	55.64%	83.35%	54.46%	68.59%
KNN	88.14%	54.46%	91.39%	58.25%	73.06%
Random forest	88.86%	53.82%	91.19%	55.31%	72.30%
AdaBoosting	88.82%	57.67%	91.65%	53.06%	72.80%
Naive bayesian	67.31%	63.33%	69.21%	55.98%	63.96%
DAC	79.85%	55.78%	82.06%	53.67%	67.84%
Averaged accuracy	82.32%	56.78%	84.81%	55.12%	

Based on the multiple features of the above two modalities of EEG and fNIRS, we then used a Normalized-ReliefF method for fusion processing and classification. From Table 3, we can see that compared with features based only on EEG and only based on fNIRS, the classification accuracy of multi-modal features based on the fusion of the Normalized-ReliefF method have been significantly improved, with an average accuracy rate of 93.38%. To further demonstrate the effectiveness of the fusion method of Normalized-ReliefF, we also conducted a set of comparative experiments, that is, comparing classification performance of the direct feature splicing method, the normalization algorithm only, the ReliefF algorithm only, and the Normalized-ReliefF method. As shown in Table 3, the average classification accuracy of the six classifiers based on the direct feature splicing method is 85.25%, while the classification accuracy of multi-modal fusion based only on the normalization algorithm is 92.64%, which is 7.39% higher than the classification accuracy based on the direct feature splicing method. In the multi-modal fusion classification experiment based only on the ReliefF algorithm, the average accuracy of the six classifications was 89.40%, which is 4.15% higher than the direct feature splicing method. Experimental results prove that these two algorithms are more effective than the direct feature splicing method. The Normalized-ReliefF algorithm, which combines the normalization and ReliefF algorithm, has the highest average classification accuracy, which is 8.43% higher than the direct stitching method, 1.02% higher than the normalized algorithm only, and 4.28% higher than the ReliefF algorithm only. These results prove that the Normalized-ReliefF method we proposed is effective for the multi-modal fusion of EEG-fNIRS. In the comparative experiment, we also calculated the running time required to classify a single subject in each classifier in four methods (i.e., direct splicing method, only the normalize algorithm, only the ReliefF algorithm, and the Normalized-ReliefF method).

**Table 3:** Classification accuracy of different classifiers based on the EEG and fNIRS fusion feature.

	**Splicing**	**Only normalization**	**Only ReliefF**	**Normalized-reliefF**
SVM	87.24%	96.84%	92.81%	97.72%
KNN	72.38%	91.55%	74.61%	92.12%
Random forest	91.63%	97.87%	98.04%	98.38%
AdaBoosting	90.22%	94.41%	94.39%	95.79%
Naive bayesian	79.43%	79.91%	80.83%	82.90%
DAC	90.62%	95.26%	95.72%	96.79%
Accuracy averaged	85.25%	92.64%	89.40%	93.68%

## Discussion

Many studies have shown that music not only has a positive effect on people's emotions, but the pleasure of music also has a positive effect on the brain's activity response. To better understand the effect of personal preferred music on brain activity, in this study, we combined EEG and fNIRS technology to simultaneously measure the brain activity of healthy subjects when listening to neutral music and their preferred music, and used the Normalized-ReliefF method to identify the characteristics of the brain activity evoked by the two types of music. Previous work exploring the influence of music on brain activity was mostly based on a single-modality, and our work is to integrate two different modalities, EEG and fNIRS, so as to obtain more abundant brain information from the two aspects of neuroelectric signals and cerebral hemodynamic signals. Furthermore, most of the previous feature fusion studies were to fuse multiple features of a single-modality such as EEG signals (Nguyen et al., 2018; Li et al., 2019; Hua et al., 2021). Our study focuses on the feature fusion of two different brain signals (EEG and fNIRS), which are different in principle, acquisition mechanism, and signal amplitude. Therefore, we combined normalization and ReliefF algorithms in the fusion strategy. Normalization is mainly used to modulate features from different modes to the same scale. The ReliefF algorithm is mainly used to optimize and select high-dimensional multi-scale features from two modalities.

From the results of EEG and fNIRS analysis, we can draw a conclusion that both personal preferred and neutral music can enhance brain activity and have similar activation patterns. The activated area is mainly distributed in the prefrontal lobe. The functions of the prefrontal lobe are mainly related to emotion, cognition and memory, and is also related to reward system of human brain (Rouault et al., 2019). There's evidence showing that music is closely connected to the stimulation of neurons and executive function of the prefrontal cortex. There may be integration of music and autobiographical memory in the medial prefrontal cortex, facilitating retrieval of personally salient episodic memories when listening to familiar musical excerpts (Janata, 2009).

In the EEG analysis, we also found that the occipital lobe was also partially activated when the subjects listened to music. Since we did not collect fNIRS data from the occipital lobe, it was impossible to compare with EEG data. The activation of the occipital lobe may due to the fact that this experiment required the subjects to close their eyes during the experiment. Participants did experiments with their eyes closed, the activation of the occipital lobes of the brain will be significantly enhanced in the α band. Barry and Blasio (2017) studied the changes in the EEG of the elderly and young people in the resting state with the eyes open and closed, and found that people have a stronger α response in the occipital lobe when the eyes are closed than when the eyes are open. Our fNIRS results show that in addition to the prefrontal lobe, the temporal lobe is also partially activated, especially the right temporal lobe, which is consistent with a previous study (Alfredson et al., 2004). The functions of temporal lobe is mainly related to hearing, memory and mental activity (Bougeard and Fischer, 2002). There is evidence that music can also activate the temporal lobe area (Alfredson et al., 2004; Hosseini and Hosseini, 2019). For the right hand, the right temporal lobe is its non-dominant hemispherical temporal lobe. Its main function is to recognize high-level neural activities such as memory, association, and comparison. Music have been demonstrated has a positive effect on cognitive development of the brain, including memory, learning and attention (Franco Jarava, 2018). Pan et al. (2018) projected the DE and common spatial pattern (CSP) features of happy and sad emotions onto the scalp for subjects and confirmed that different emotional states will produce different responses in brain regions. At the same time, they also found that during positive emotion processing, the neural patterns had significantly higher brain responses at the temporal lobes.

How to integrate information of different modalities has always been a difficult problem in the field of multi-modal research. In this study, we proposed an EEG-fNIRS multi-modal integration framework from data collection to data analysis to explore the effect of personal preference music on brain activity. First, we used a synchronous trigger device in data acquisition to integrate EEG and fNIRS into an EEG-fNIRS synchronous acquisition system, achieving the synchronous recording of two different brain signals. In data analysis, in order to make full use of the information of the two modalities, we proposed an improve Normalized-ReliefF method to fuse and optimize multi-modal features. Most of the current studies are to splice the features of different modalities directly into the classifier without any processing (Al-Shargie et al., 2016; Hong et al., 2018; Cicalese et al., 2020). Although this splicing method can also achieve higher accuracy than single-modalities, it does not consider that the types and scales of features from different modes are different, and there is also information redundancy between them. Obviously, this processing method cannot make full and effective use of multi-modal information and truly realize the superiority of multi-modal systems.

The Normalized-ReliefF method we proposed can effectively solve the problem of feature fusion of EEG and fNIRS. We first normalized all the feature data of EEG and fNIRS to modulate them to the same scale. Then, the ReliefF method was used to perform feature selection on multiple features from different modalities and different dimensions to achieve the effect of removing redundant information and reducing dimensionality at the same time. The normalization process can not only evaluate the features of the two modalities of EEG and fNIRS on the same scale, but also shorten the data training time to a certain extent. The ReliefF algorithm is a feature weighting algorithm, which assigns different weights to features according to the correlation of each feature and category, and features with a weight less than a certain threshold will be removed The running time of the ReliefF algorithm increases linearly with the increase in the number of samples and the number of original features, so the running efficiency is relatively high (Stamate et al., 2018; Kshirsagar and Kumar, 2021; Satapathy and Loganathan, 2021). In our previous work (Pan et al., 2021), we also confirmed the effectiveness of the ReliefF algorithm for EEG-based multi-modal feature extraction, and it can achieve the effect of feature optimization and feature dimensionality reduction to a certain extent. The Normalized-ReliefF method we proposed can effectively solve the problem of fusion of EEG and fNIRS features.

In order to further verify the effectiveness of the Normalized-ReliefF method, we conducted a set of comparative ablation experiments. By comparing the classification performance of direct feature splicing method, only the normalized algorithm, only the ReliefF algorithm and the Normalized-ReliefF, we found that the Normalized-ReliefF method can effectively improve the average accuracy of the classification of brain activities induced by preferred and neutral music. In terms of calculation time, the Normalized-ReliefF method is also more efficient than the other three fusion methods. This fully proves the efficiency of the Normalized-ReliefF method in the fusion of the two modal features of EEG and fNIRS. Furthermore, our experimental results also verify that the multi-modal features based on EEG-fNIRS have better classification performance than signal-modal features based only on EEG and only on fNIRS, because the combination of the two modalities can provide richer brain activity information.

In the exploration of the effect of preferred music on brain activity in this work, we proposed an EEG-fNIRS integration framework to systematically integrate EEG-fNIRS multi-modal signals from data collection to data analysis. The results of multi-modal analysis found that personally preferred music promotes brain activity, and our proposed fusion method, Normalized-ReliefF, effectively improves the recognition accuracy of brain activity induced by different music. Our work may provide neuroimaging-based references for the research and application of personalized music therapy. Taking patients with DOC as an example, patients with DOC can be divided into MCS and VS patients (Xie et al., 2018). At present, the clinical diagnosis and assessment of consciousness in patients with DOC mainly rely on behavioral scales, but the misdiagnosis rate of this method is as high as 37–43% (Hirschberg and Giacino, 2011). It is very necessary to make an objective and effective diagnosis of the consciousness of patients with consciousness disorders. Our work has confirmed that personal preferred music can promote the brain. In other words, personal preferred music cannot only be used as a brain stimulus, but also as a treatment method for patients. In view of the current difficulties in diagnosing and treating DOC patients in clinical practice, we can use the patients' personal preferred music as a stimulus, and explore the characteristics of brain activity induced by music in patients with different states of consciousness by using multi-modal imaging technology.

However, our study still has some limitations. First, our sample size is relatively small. Secondly, our experimental paradigm only listened to preferred music and neutral music once, and did not conduct multiple rounds of repeated task. Finally, we only analyze the activation analysis, not the analysis of the brain functional connectivity. In the future, we will increase samples, improve the experimental design and data analysis methods, and include patients with neurological diseases (such as DOC patients) into the scope of subjects to compare the effects of personal preferred music on healthy subjects and patients' brain functions, and truly realize music therapy.

## Conclusion

In this study, we proposed the integration framework of the two modalities of EEG and fNIRS, including data collection and various data processing and analysis. We used this multi-modal integration framework to explore the characteristics of brain activity induced by personal preferred and neutral music, and found that music can enhance the brain activity, especially the prefrontal lobe, and personal preferred music activated their brain more than neutral music. We also proposed an improved Normalized-ReliefF algorithm to fuse and optimize multiple features of two different physiological signals EEG and fNIRS to identify the characteristics of brain activity induced by personal preference music and neutral music. We found that using the Normalized-ReliefF algorithm is more effective than the method of simple multi-feature vector splicing of the two modalities for classification. We also found that the classification accuracy rates obtained by using fusion features based on EEG-fNIRS are higher than the classification accuracy rates obtained by EEG-based features and fNIRS-based features, which proves that multi-modal brain imaging can provide better classification performance than single-modality. Our work can provide an objective reference based on neuroimaging for the research and application of personalized music therapy.

## Data Availability Statement

The raw data supporting the conclusions of this article will be made available by the authors, without undue reservation.

## Ethics Statement

The studies involving human participants were reviewed and approved by Ethics Committee of South China Normal University. The patients/participants provided their written informed consent to participate in this study.

## Author Contributions

LQ, YZ, and JP: conceptualization and writing-review and editing. LQ, YZ, and ZH: methodology. YZ: software, data curation, and writing-original draft preparation. LQ, ZH, XW, YC, and CZ: validation. LQ, YZ, ZH, and JP: formal analysis. LQ, QX, ZH, XW, YC, CZ, and JP: investigation. LQ, YZ, QX, XW, YC, and CZ: resources. LQ, YZ, ZH, XW, YC, and JP: visualization. LQ and JP: supervision and funding acquisition. LQ, QX, and CZ: project administration. All authors have read and agreed to the published version of the manuscript.

## Funding

This work was supported by the Guangdong Basic and Applied Basic Research Foundation under grant 2019A1515110388, the Key Realm R&D Program of Guangzhou under grant 202007030005, and the National Natural Science Foundation of China under grants 62076103, 81974154, and 82171174.

## Conflict of Interest

The authors declare that the research was conducted in the absence of any commercial or financial relationships that could be construed as a potential conflict of interest.

## Publisher's Note

All claims expressed in this article are solely those of the authors and do not necessarily represent those of their affiliated organizations, or those of the publisher, the editors and the reviewers. Any product that may be evaluated in this article, or claim that may be made by its manufacturer, is not guaranteed or endorsed by the publisher.

## References

[B1] Åkerstedt T. Gillberg M. (1986). Sleep duration and the power spectral density of the EEG. Electroencephalogr. Clin. Neurophysiol. 64, 119–122. 10.1016/0013-4694(86)90106-9 2424728

[B2] Alfredo R. (2015). Music therapy interventions in Parkinson's Disease: the state-of-the-art. Front. Neurol. 6, 185. 10.3389/fneur.2015.00185 26379619 PMC4553388

[B3] Alfredson B. B. Risberg J. Hagberg B. Gustafson L. (2004). Right temporal lobe activation when listening to emotionally significant music. Appl. Neuropsychol. 11, 161–166. 10.1207/s15324826an1103_4 15590350

[B4] Al-Shargie F. M. Tang T. B. Badruddin N. Dass S. C. Kiguchi M. (2016). Mental Stress Assessment Based on Feature Level Fusion of fNIRS and EEG Signals, in 2016 6th International Conference on Intelligent and Advanced Systems (ICIAS) - 6th International Conference on Intelligent and Advanced Systems (Kuala Lumpur), 1–5.

[B5] Barry R. J. Blasio F. D. (2017). EEG differences between eyes-closed and eyes-open resting remain in healthy ageing. Biol. Psychol. 129, 293. 10.1016/j.biopsycho.2017.09.010 28943465

[B6] Bennet A. Bennet D. H. (2008). The human knowledge system: music and brain coherence. Vine 38, 277–295. 10.1108/03055720810904817

[B7] Bigliassi M. León-Domínguez U. Altimari L. R. (2015). How does the prefrontal cortex “listen” to classical and techno music? A functional near-infrared spectroscopy (fNIRS) study. Psychol. Neurosci. Methods 8, 246–256. 10.1037/h0101064

[B8] Blood A. J. Zatorre R. J. Bermudez P. Evans A. C. (1999). Emotional responses to pleasant and unpleasant music correlate with activity in paralimbic brain regions. Nat. Neurosci. 2, 382–387. 10.1038/7299 10204547

[B9] Bougeard R. Fischer C. (2002). The role of the temporal pole in auditory processing. Epileptic Disord. 4(Suppl. 1), S29. 10.7227/CE.78.1.7 12424088

[B10] Carrière M. Larroque S. K. Charlotte C. Bahri M. A. Heine L. (2020). An echo of consciousness: brain function during preferred music. Brain Connect. 10:385–395. 10.1089/brain.2020.0744 32567335

[B11] Chen X. Wei Q. Jing R. Fan Y. (2021). Effects of music therapy on cancer-related fatigue, anxiety, and depression in patients with digestive tumors: a protocol for systematic review and meta-analysis. Medicine 100, e25681. 10.1097/MD.0000000000025681 34087821 PMC8183835

[B12] Chincarini M. Costa E. D. Qiu L. Spinelli L. Torricelli A. (2020). Reliability of fNIRS for noninvasive monitoring of brain function and emotion in sheep. Sci. Rep. 10, 14726. 10.1038/s41598-020-71704-5 32895449 PMC7477174

[B13] Cicalese P. A. Li R. Ahmadi M. B. Wang C. Francis J. T. Selvaraj S.. (2020). An EEG-fNIRS hybridization technique in the four-class classification of alzheimer's disease. Neurosci. Methods 336. 10.1016/j.jneumeth.2020.108618 32045572 PMC7376762

[B14] Duan R. N. Zhu J. Y. Lu B. L. (2013). Differential entropy feature for EEG-based emotion classification, in International IEEE/EMBS Conference on Neural Engineering (Seattle, WA), 81–84.

[B15] Franco Jarava J. (2018). Preferred Music Effect on Human Brain Using Functional Near-Infrared Spectroscopy. Barcelona: Universitat Politècnica de Catalunya.

[B16] Geethanjali B. Adalarasu K. Mohan J. Seshadri N. (2018). Music induced brain functional connectivity using EEG sensors: a study on indian music. IEEE Sens. J. 19:1. 10.1109/JSEN.2018.287340227295638

[B17] Gerdner L.A. (2000). Effects of individualized versus classical “relaxation” music on the frequency of agitation in elderly persons with Alzheimer's disease and related disorders. Int. Psychogeriatr. 12, 49–65. 10.1017/S1041610200006190 10798453

[B18] Greenberg D. M. Baron-Cohen S. Stillwell D. J. Kosinski M. Rentfrow P. J. (2015). Musical preferences are linked to cognitive styles. PLoS ONE 10:e0131151. 10.1371/journal.pone.0131151 26200656 PMC4511638

[B19] Gui R. Chen T. Nie H. (2019). The impact of emotional music on active ROI in patients with depression based on deep learning: a task-state fMRI study. Comp. Intell. Neurosci. 2019, 1–14. 10.1155/2019/5850830

[B20] He Z. Li Z. Yang F. Wang L. Pan J. (2020). Advances in multimodal emotion recognition based on brain-computer interfaces. Brain Sci. 10, 687. 10.3390/brainsci10100687 33003397 PMC7600724

[B21] Hirschberg R. Giacino J. T. (2011). The vegetative and minimally conscious states: diagnosis, prognosis and treatment. Neurol. Clin. 29, 773–786. 10.1016/j.ncl.2011.07.009 22032660

[B22] Hong K. S. Khan M. J. Hong M. J. (2018). Feature extraction and classification methods for hybrid fNIRS-EEG brain-computer interfaces. Front. Hum. Neurosci. 12, 246. 10.3389/fnhum.2018.00246 30002623 PMC6032997

[B23] Hosseini E. Hosseini S. A. (2019). Therapeutic effects of music: a review. RepHealth Care 4, 1–13.

[B24] Hua Y. Zhong X. Zhang B. Yin Z. Zhang J. (2021). Manifold feature fusion with dynamical feature selection for cross-subject emotion recognition. Brain Sci. 11, 1392. 10.3390/brainsci11111392 34827391 PMC8615971

[B25] Hubert B. Rishabh G. Falk T. H. (2017). Mental task evaluation for hybrid NIRS-EEG brain-computer interfaces. Comp Intell. 2017, 1–24. 10.1155/2017/3524208 29181021 PMC5664195

[B26] Jagiello R. Pomper U. Yoneya M. Zhao S. Chait M. (2019). Rapid brain responses to familiar vs. unfamiliar music – an EEG and pupillometry study. Sci. Rep. 9:15570. 10.1038/s41598-019-51759-9 31666553 PMC6821741

[B27] James R. Sigafoos J. Green V. A. Lancioni G. E. O'Reilly M. F. Lang R.. (2015). Music therapy for individuals with autism spectrum disorder: a systematic review. Rev. J. Autism Dev. Disord. 2, 39–54. 10.1007/s40489-014-0035-4

[B28] Janata P. (2009). The neural architecture of music-evoked autobiographical memories. Cerebral Cortex 19, 2579–2594. 10.1093/cercor/bhp008 19240137 PMC2758676

[B29] Jared B. Yuya T. Shinya K. Koichi Y. (2018). Spectral-spatial differentiation of brain activity during mental imagery of improvisational music performance using MEG. Front. Hum. Neurosci. 12, 156. 10.3389/fnhum.2018.00156 29740300 PMC5928205

[B30] Khan M. U. Hasan M. A. H. (2020). Hybrid EEG-fNIRS BCI fusion using multi-resolution singular value decomposition (MSVD). Front. Hum. Neurosci. 14, 599802. 10.3389/fnhum.2020.599802 33363459 PMC7753369

[B31] Koelsch S. (2015). Music-evoked emotions: principles, brain correlates, and implications for therapy. Ann. N. Y. Acad. Sci. 1337, 193–201. 10.1111/nyas.12684 25773635

[B32] Kshirsagar D. Kumar S. (2021). An efficient feature reduction method for the detection of DoS attack. ICT Exp. 7:371–375. 10.1016/j.icte.2020.12.006

[B33] Li P. Y. Liu H. Si Y. Li C. Li F. Zhu X.. (2019). EEG based emotion recognition by combining functional connectivity network and local activations. IEEE Transact. Biomed. Eng. 1:2869–2881. 10.1109/TBME.2019.2897651 30735981

[B34] Li R. Zhao C. Wang C. Wang J. Zhang Y. (2020a). Enhancing fNIRS analysis using EEG rhythmic signatures: an EEG-informed fNIRS analysis study. IEEE Transact. Biomed. Eng. 67, 2789–2797. 10.1109/TBME.2020.2971679 32031925

[B35] Li Z. Qiu L. Li R. He Z. Xiao J. Liang Y.. (2020b). Enhancing BCI-based emotion recognition using an improved particle swarm optimization for feature selection. Sensors 20:3028. 10.3390/s20113028 32471047 PMC7309000

[B36] Meziani A. Djouani K. D. Medkour T. Chibani A. (2019). A Lasso quantile periodogram based feature extraction for EEG-based motor imagery. J. Neurosci. Methods 328, 108434. 10.1016/j.jneumeth.2019.108434 31569036

[B37] Murrock C. J. Higgins P. A. (2010). The theory of music, mood and movement to improve health outcomes. J. Adv. Nurs. 65, 2249–2257. 10.1111/j.1365-2648.2009.05108.x 20568327 PMC3573365

[B38] Naseer N. Hong K. S. (2015). fNIRS-based brain-computer interfaces: a review. Front. Hum. Neurosci. 9, 3. 10.3389/fnhum.2015.00003 25674060 PMC4309034

[B39] Nguyen D. Nguyen K. Sridharan S. Dean D. Fookes C. (2018). Deep spatio-temporal feature fusion with compact bilinear pooling for multimodal emotion recognition. Comp. Vis. Image Understand. 174, 33–42. 10.1016/j.cviu.2018.06.005

[B40] Niet G. D. Tiemens B. Lendemeijer B. Hutschemaekers G. (2010). Music-assisted relaxation to improve sleep quality: meta-analysis. J. Adv. Nurs. 65, 1356–1364. 10.1111/j.1365-2648.2009.04982.x 19456998

[B41] Pan J. Xie Q. Huang H. He Y. Sun Y. Yu R.. (2018). Emotion-related consciousness detection in patients with disorders of consciousness through an EEG-based BCI system. Front. Hum. Neurosci. 12, 198. 10.3389/fnhum.2018.00198 29867421 PMC5962793

[B42] Pan J. Xie Q. Qin P. Chen Y. He Y. Huang H.. (2020). Prognosis for patients with cognitive motor dissociation identified by brain-computer interface. Brain 143, 1–13. 10.1093/brain/awaa026 32101603 PMC7174053

[B43] Pan J. Zhang J. Wang F. Liu W. Huang H. Tang W.. (2021). Automatic sleep staging based on EEG-EOG signals for depression detection. Intell. Automat. Soft Comp. 28, 53–71. 10.32604/iasc.2021.015970

[B44] Putze F. Hesslinger S. Tse C. Y. Huang Y. Y. Schultz T. (2013). Hybrid fNIRS-EEG based classification of auditory and visual perception processes. Front. Neurosci. 8, 373. 10.3389/fnins.2014.00373 25477777 PMC4235375

[B45] Qi S. Calhoun V. D. Erp T. V. Bustillo J. Damaraju E. Turner J. A.. (2018). Multimodal fusion with reference: searching for joint neuromarkers of working memory deficits in schizophrenia. IEEE Trans. Med. Imaging 37, 1. 10.1109/TMI.2017.2725306 28708547 PMC5750081

[B46] Quiroga R. Q. Rosso O. A. Başar E. Schürmann M. (2001). Wavelet entropy in event-related potentials: a new method shows ordering of EEG oscillations. Biol. Cybern. 84, 291–299. 10.1007/s004220000212 11324340

[B47] Reschke-Hernández A. E. Belfi A. M. Guzmán-Vélez E. Tranel D. (2020). Hooked on a feeling: influence of brief exposure to familiar music on feelings of emotion in individuals with Alzheimer's Disease. J. Alzheimers Dis. 78, 1019–1031. 10.3233/JAD-200889 33074236

[B48] Rollnik J. D. Eckart A. (2014). Music in disorders of consciousness. Front. Neurosci. 8, 190. 10.3389/fnins.2014.00190 25071434 PMC4080825

[B49] Rouault M. Drugowitsch J. Koechlin E. (2019). Prefrontal mechanisms combining rewards and beliefs in human decision-making. Nat. Commun. 10:301. 10.1038/s41467-018-08121-w 30655534 PMC6336816

[B50] Sarkamo T. Tervaniemi M. Laitinen S. Forsblom A. Soinila S. Mikkonen M.. (2008). Music listening enhances cognitive recovery and mood after middle cerebral artery stroke. Brain Sci. 131 (Pt 3), 866–876. 10.1093/brain/awn013 18287122

[B51] Satapathy S. K. Loganathan D. (2021). A study of human sleep stage classification based on dual channels of EEG signal using machine learning techniques. SN Comp. Sci. 2:157. 10.1007/s42979-021-00528-5

[B52] Stamate D. Alghambdi W. Ogg J. Hoile R. Murtagh F. (2018). A machine learning framework for predicting dementia and mild cognitive impairment, in 2018 17th IEEE International Conference on Machine Learning and Applications (ICMLA) (Orlando, FL), 671–678.

[B53] Witte M. D. Pinho S. Stams G. J. Moonen X. Hooren S. V. (2020). Music therapy for stress reduction: a systematic review and meta-analysis. Health Psychol. Rev. 14, 1–26. 10.1080/17437199.2020.1846580 33176590

[B54] Xie Q. Pan J. Chen Y. He Y. Ni X. Zhang J.. (2018). A gaze-independent audiovisual brain-computer Interface for detecting awareness of patients with disorders of consciousness. BMC Neurol. 18:144. 10.1186/s12883-018-1144-y 30296948 PMC6176505

[B55] Yücel M. Lühmann A. Scholkmann F. Gervain J. Wolf M. J. N. (2021). Best practices for fNIRS publications. Neurophotonics. 8. 10.1117/1.NPh.8.1.012101 33442557 PMC7793571

[B56] Zheng M. Lin H. Chen F. (2020). An fNIRS study on the effect of music style on cognitive activities, in 2020 42nd Annual International Conference of the IEEE Engineering in Medicine and Biology Society (EMBC) in Conjunction With the 43rd Annual Conference of the Canadian Medical and Biological Engineering Society. 10.1109/EMBC44109.2020.9176441 33018685

